# Consistency of *BRCA1* and *BRCA2* Variant Classifications Among Clinical Diagnostic Laboratories

**DOI:** 10.1200/PO.16.00020

**Published:** 2017-04-11

**Authors:** Stephen E. Lincoln, Shan Yang, Melissa S. Cline, Yuya Kobayashi, Can Zhang, Scott Topper, David Haussler, Benedict Paten, Robert L. Nussbaum

**Affiliations:** **Stephen E. Lincoln**, **Shan Yang**, **Yuya Kobayashi**, and **Scott Topper**, Invitae; **Robert L. Nussbaum**, University of California, San Francisco, San Francisco; and **Melissa S. Cline**, **Can Zhang**, **David Haussler**, and **Benedict Paten**, University of California, Santa Cruz, Santa Cruz, CA.

## Abstract

**Purpose:**

Genetic tests of cancer predisposition genes, *BRCA1* and *BRCA2*, inform significant clinical decisions for both physicians and patients. Most uncovered variants are benign, and determining which few are pathogenic—disease causing—is sometimes challenging and can potentially be inconsistent among laboratories. The ClinVar database makes deidentified clinical variant classifications from multiple laboratories publicly available for comparison and review, per recommendations by the American Medical Association, the American College of Medical Genetics, the National Society for Genetic Counselors, and other organizations.

**Methods:**

Classifications of more than 2,000 *BRCA1/2* variants in ClinVar that represent approximately 22,000 patients were dichotomized as clinically actionable or not actionable and compared among as many as seven laboratories. The properties of these variants and classification differences were investigated in detail.

**Results:**

Per-variant concordance was 98.5% (CI, 97.9% to 99.0%). All discordant variants were rare; thus, per-patient concordance was estimated to be higher (99.7%). ClinVar facilitated resolution of many of the discordant variants, and concordance increased to 99.0% per variant and 99.8% per patient when reclassified, but not yet resubmitted, variants and submission errors were addressed. Most of the remaining discordances seemed to involve either legitimate differences in expert judgment regarding particular scientific evidence or were classifications that predated the availability of important scientific evidence.

**Conclusion:**

Significant classification disagreements among professional clinical laboratories represented in ClinVar are infrequent yet important. Unrestricted sharing of clinical genetic data allows detailed interlaboratory quality control and peer review, as exemplified by this study.

## INTRODUCTION

Hereditary breast and ovarian cancer is a cancer predisposition syndrome that results from inherited—that is, germline—loss-of-function mutations in *BRCA1* or *BRCA2* genes, collectively, *BRCA1/2*. Such pathogenic, or disease-causing, genetic variants result in a 40% to 80% lifetime risk of developing breast cancer, an 11% to 40% risk of ovarian cancer, and striking increases in the risk of male breast, pancreatic, and prostate cancers.^1,2^ Up to 10% of breast cancers are caused by these genes.^3,4^ Approximately one in 250 individuals of European descent are born with a pathogenic variant in *BRCA1/2*, and prevalence is much higher in certain populations—for example, Ashkenazi Jews.^5,6^

Decades of clinical testing and research have uncovered tens of thousands of *BRCA1/2* genetic variants across the human population.^7^ The great majority of these variants are benign and confer no increased cancer risk, whereas others are pathogenic. Still others are considered variants of uncertain significance (VUS) when the current scientific evidence for or against pathogenicity is inadequate or conflicting. To help standardize variant interpretation, the American College of Medical Genetics (ACMG) and the Association for Molecular Pathology (AMP) jointly issued revised guidelines^8^ for variant classification. Although more comprehensive and specific than earlier guidelines,^9,10^ these guidelines still require laboratory directors to use expert judgment in evaluating the quality of available evidence. Thus, the classifications of some genetic variants may vary among laboratories. Moreover, scientific evidence continually evolves, which can change the classifications of some variants over time. Whereas VUS, likely benign, and benign variants in *BRCA1*/*2* are not medically actionable, pathogenic and likely pathogenic variants are actionable, which warrants consideration of additional screening, prevention, or treatment options.^6,11,12^ Thus, rigorous and consistent variant interpretation is critical to patient care.

Variant classifications can also potentially conflict if one laboratory has access to proprietary data that are unavailable to others. In the 1990s, Myriad Genetics patented the *BRCA1*/*2* genes and prohibited testing by other laboratories.^13,14^ Myriad Genetics continued as the sole provider for nearly 20 years until the patents were overturned. The company used its monopoly to accumulate a substantial database of variants that it ceased releasing publicly in 2006 and from which it claims a competitive advantage.^15-17^ This practice is contrary to the recommendations of the American Medical Association (AMA), the ACMG, the National Society for Genetic Counselors, and other organizations.^18-20^ Recognizing that shared knowledge about genetic variants is critical to high-quality medical care, the National Institutes of Health established ClinVar, a public database of clinically observed genetic variants, their pathogenicity classifications from various laboratories, and a summary of the scientific evidence used in those classifications.^21-25^ Whereas many commercial and academic laboratories collaboratively submit data to ClinVar, others, including Myriad, do not. Nevertheless, a substantial Myriad Genetics data set has been submitted by ordering clinicians and patients through the Sharing Clinical Reports Project (SCRP).^13,26^ In this study, we used publicly available data from ClinVar to assess agreement among clinical laboratories for classifications of *BRCA1*/*2* variants.

## METHODS

Classifications of *BRCA1/2* variants were extracted from the ClinVar May 2016 release. Variants in ClinVar are classified as pathogenic, likely pathogenic, VUS, likely benign, or benign, which is consistent with ACMG/AMP terminology.^8^ Laboratory-specific classification categories—for example, deleterious instead of pathogenic or polymorphism instead of benign—are mapped to the standardized nomenclature. Our inclusion criteria restricted analysis to data from licensed clinical laboratories with at least 200 classified *BRCA1/2* variants in ClinVar, among which most (> 50%) were less than 5 years old. We thus excluded data from research laboratories, consortia, smaller—possibly less experienced—clinical laboratories, and older data sets. ClinVar submissions that we knew were pending, that is, provided to ClinVar but not yet merged into a monthly release, were incorporated. Duplicate entries were identified and merged, and clearly erroneous entries were repaired or removed (Data Supplement). The complete data set that was used in our analysis is also provided as a Data Supplement.

To compare potential clinical impact, we dichotomized classifications into positive (pathogenic, likely pathogenic) or not positive (benign, likely benign, VUS). Although many laboratories exclude benign and likely benign variants from clinical reports, these variants are often submitted to ClinVar and many are available for comparison.^27^ SCRP, which is derived directly from clinical reports—and thus benign and likely benign variants are under-represented from this submitter (Table 1 and Data Supplement)—is an exception.

**Table 1.**
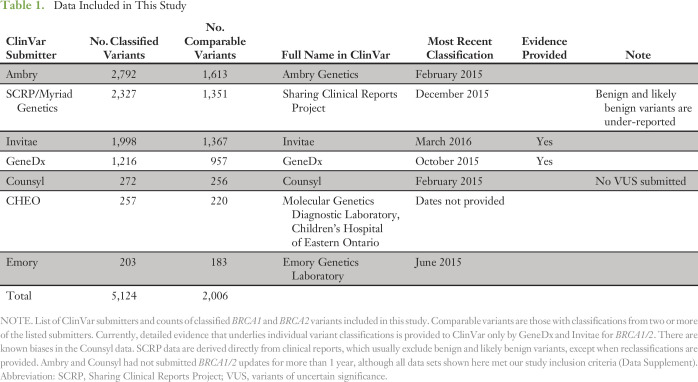
Data Included in This Study

General population allele frequencies for these variants were determined by using the ExAC database,^28^ the 1000 Genomes Project phase III database,^29^ and the Exome Variant Server.^30^ Common variants were defined as those with allele frequencies greater than 1% in any of these databases. We used a separate sequential series of more than 30,000 patients who were clinically tested for *BRCA1/2* to measure allele frequencies in a clinical population. These patients’ variants were also part of the ClinVar data set described above.

CIs were computed by using the Wilson method.^31^ Evaluation of the scientific evidence that underlies discordant classifications was performed according to the most recent ACMG/AMP recommendations.^8^ Our methods for estimating the number of patients who were expected to have discordant variants are detailed in the Data Supplement.

## RESULTS

**Fig 1:**
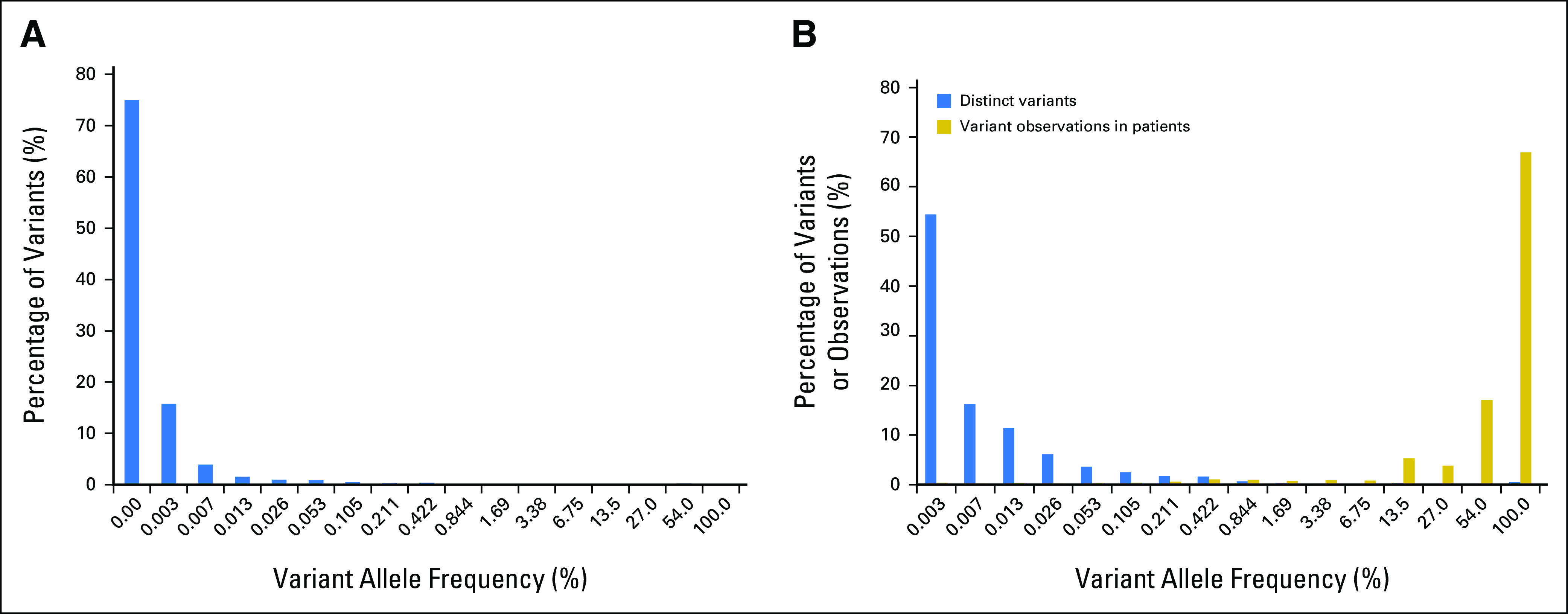
Histograms of ClinVar variants (A) by population allele frequency in ExAC and (B) by prevalence in our clinical database. By either measure, most variants in ClinVar are rare, although the vast majority of variants observed in patients are repeated occurrences of a small number of common and intermediate frequency variants.

**Fig 2:**
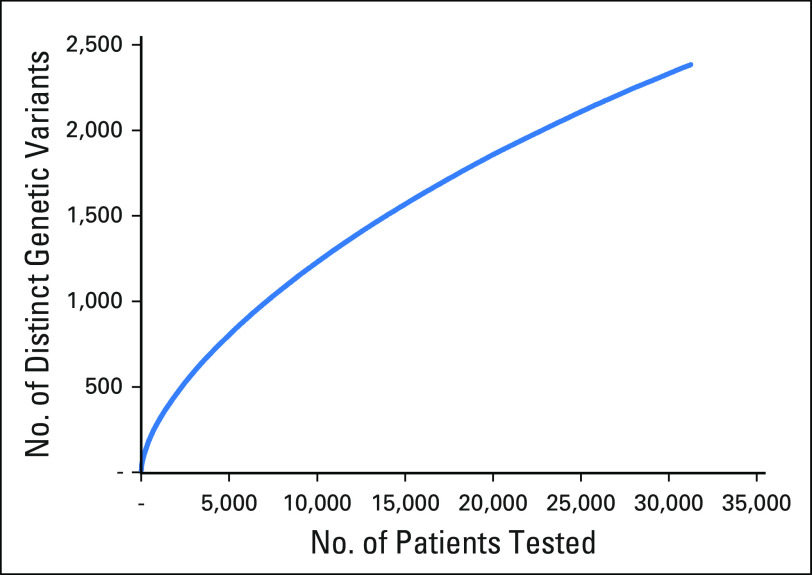
Total number of distinct genetic variants observed as patients were added to the clinical database. Because many variants are rare, new variants continue to be accumulated even after many patients have been sequenced. From these data, we estimate that the comparable ClinVar variants in this study (n = 2,006) correspond to the number that would be observed if approximately 22,000 patients had been tested by the same laboratories.

There were 5,124 *BRCA1/2* variants submitted to ClinVar by seven groups that met our inclusion criteria (Table 1). Of these variants, 2,006 had classifications from two or more laboratories that were available for comparison. We call these comparable variants (Data Supplement). The remaining variants had been submitted by only a single source. Nearly 90% of these variants (1,769 of 2,006) were rare, having allele frequencies less than 0.05% in all of the general population databases we examined and less than 0.1% in our clinical database (Fig 1). We estimate that comparable variants represent testing of approximately 22,000 patients (Fig 2). Comparable variants were a representative subset of 5,124 ClinVar variants in overall properties, with an expected bias away from rare variants, albeit small in magnitude (Table 2). ClinVar data were also representative of those observed in clinical practice, with some submitter-specific exceptions (Table 1 and Data Supplement).

**Table 2.**
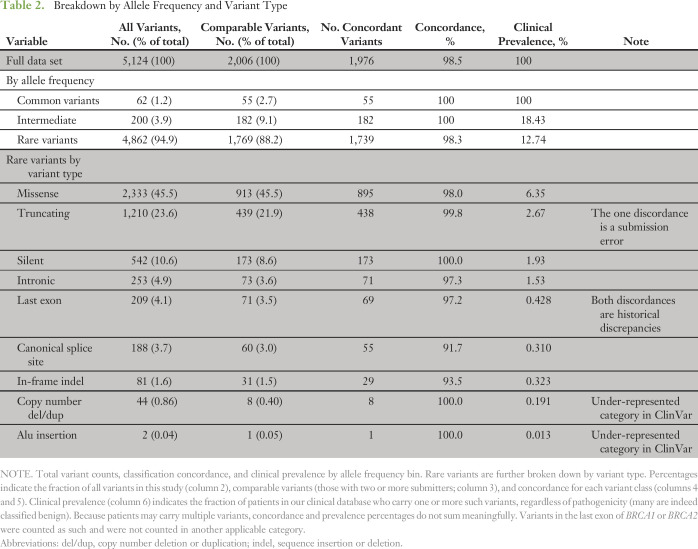
Breakdown by Allele Frequency and Variant Type

We compared variant classifications in terms of whether they would or would not potentially affect clinical management (see Methods). On a per-variant basis, we found high concordance: 98.5% of comparable variants (1,976 of 2,006; CI, 97.9% to 99.0%) had concordant classifications among all submitters. Only 30 of 2,006 showed discordance between any two submitters (Data Supplement). Pairwise concordance between laboratories was also high, varying between 97.2% and 100.0% (Data Supplement).

**Fig 3:**
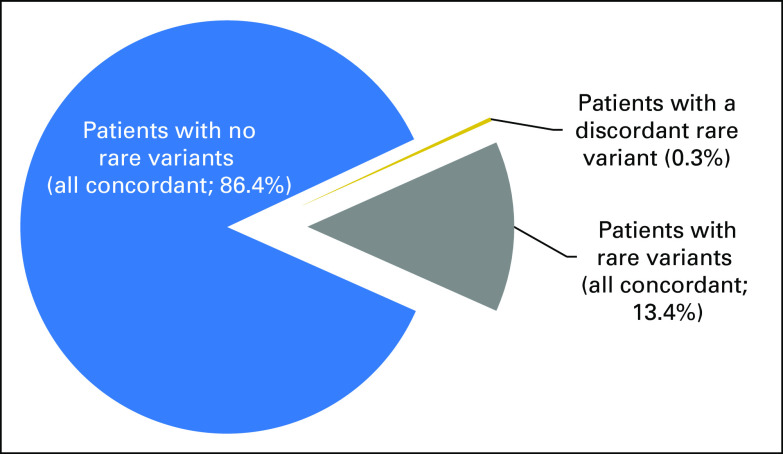
Summary of per-patient concordance. The only classification discordances we observed were in rare variants, which few patients carry, and most rare variants were completely concordant when observed by multiple laboratories (Data Supplement).

Of importance, the 30 clinically significant discordances were in rare variants that, by definition, are present in few patients. On the basis of the prevalence distribution of variants in clinical testing, we calculated the expected concordance on a per-patient basis to be 99.7% (Fig 3; Data Supplement). An independent calculation on the basis of population allele frequencies confirmed this result. This concordance rate is similar to that reported (99.8%) in a prior study of approximately 1,000 prospectively accrued patients that compared ACMG/AMP-based classifications with those from Myriad Genetics.^32,33^

A feature of ClinVar is that it records the date on which each classification was made, thereby allowing us to consider whether the high concordance we observed could be a result of laboratories being overly influenced by each other’s prior classifications. Such influence would be most concerning in the case of Myriad Genetics classifications submitted by SCRP, for which underlying evidence is unavailable for other laboratory directors to evaluate.^14^ We saw no evidence of such bias, as 99.4% (503 of 506) of classifications that predated a Myriad Genetics/SCRP entry were concordant with it compared with 99.1% (2,385 of 2,406) that postdated it (Data Supplement). These rates are not significantly different.

Although classifications from the ENIGMA consortium (Evidence-Based Network from the Interpretation of Germline Mutant Alleles)^34^ were not considered in our comparison, they can provide evidence that clinical laboratories use in their classifications. ENIGMA classifications were available for 250 variants in our study with, in total, 996 laboratory classifications (Data Supplement). Only two of 996 were discordant with ENIGMA, both of which were Myriad Genetics/SCRP classifications that predate the corresponding ENIGMA submissions.

Among laboratories, the largest class of discordant variants we observed were rare missense changes (18 of 30) that alter only a single amino acid in the resulting protein. These variants are numerous, comprising almost one half (913 of 2,006; 45.5%) of our data set, and the vast majority (895 of 913; 98.0%) had concordant classifications among all submitters (Table 2). Although clearly important, rare missense variants are infrequently observed in patients—6.3% prevalence in our clinical data set. Rare protein truncating and silent variants were also numerous (439 of 2,006 and 173 of 2,006, respectively; 30.5% of the data set together) and were concordantly classified with one exception. Other discordant variants were in canonical RNA splice sites (five of 30) or an intron (two of 30) or were in-frame deletions (two of 30). Relatively few variants of these types were reported; they are of low prevalence and most are concordant. Finally, two truncating mutations in the last coding exon of *BRCA2* had discordant classifications.

To gain insight into the basis for the small number of discordant classifications, we examined all publicly available evidence for and against pathogenicity for each of the 30 discordant variants. We also contacted submitting laboratories regarding specific discordant classifications, particularly those for variants with three or more submitters. We found two common explanations for discordant classifications (Data Supplement): In seven variants, there was a historical difference, meaning that one or more classifications in ClinVar was out of date, and although the variant had been reclassified, thus becoming concordant, updates had not yet been submitted to ClinVar. Two data submission errors were also identified. Including the updates to these nine variants raised concordance to 99.0% per variant and 99.8% per patient and resolved all discordant truncating mutations. In four additional cases, we suspect a historical difference because key evidence—for example, a publication—that could significantly affect classification postdates a discordant ClinVar entry. Seventeen discordances seemed to be legitimate differences in the judgment of laboratory experts who assessed available evidence of pathogenicity. Whereas proprietary data may have contributed to some discordances, particularly those that involved Myriad Genetics/SCRP data, most of those cases have an alternative, plausible explanation—that is, an historical discrepancy or difference in expert judgment.

An unusually challenging example of discordance is *BRCA1* splicing variant, c.594-2A>C, which was reported by Myriad Genetics/SCRP as pathogenic but later downgraded to VUS.^35^ Other laboratories in our study also classify this variant as VUS, though one reclassification—to VUS—had not yet propagated into ClinVar (O. Jarinova, personal communication, June 2016). We thus considered this an historical discordance. This mutation causes upregulation of an endogenous alternate RNA isoform missing exons 9 and 10 but that seems to provide *BRCA1* functionality.^36^ This result suggests that laboratory directors should carefully evaluate other mutations in exons 9 and 10 and highlights the complexities of variant classification that laboratory directors must consider in some cases.

Overall, however, our analysis suggests that a high level of concordance should, perhaps, have been expected. Most (83%) pathogenic variants were of types that are relatively straightforward to classify, for example, truncating mutations or large deletions in most regions of *BRCA1*/*2* (Data Supplement). Other variant types—notably rare missense and splice-site changes—require additional experimental or genetic data to classify, but relatively few patients (< 7%) carry any such variants, and they are often (97.6%) concordant across laboratories (Table 2). When not considered VUS, these variants are usually classified as benign, or likely benign, and not pathogenic (Data Supplement). Indeed, a comparison of ClinVar releases over the past 2 years shows that most (94.5%) missense VUS, when reclassified, are downgraded to benign or likely benign and thus remain not clinically actionable, which is consistent with prior studies.^37^

## DISCUSSION

In this study, we analyzed publicly available data from the ClinVar database and found remarkably few clinically significant discordances in the classifications of more than 2,000 variants in two well-characterized cancer risk genes, *BRCA1* and *BRCA2.* The observation that all discordant variants were rare, although most rare variants remained concordant, suggests that roughly one of 500 patients would be expected to receive results that would significantly change clinical management from the various laboratories in this study. By comparison, concordance can be far lower among pathologists who read breast biopsies or radiologists who review mammograms.^38-41^ These genetic test reports would not always be identical, both because our analysis grouped classifications—that is, we considered potentially actionable pathogenic and likely pathogenic classifications together, and we considered nonactionable benign, likely benign, and VUS together—and because laboratories vary on whether benign and likely benign variants are included in reports. VUS may also be excluded from reports in a screening context. Nevertheless, the reports’ significance for clinical management decisions remain similar.

Furthermore, we explored the likely cause of the few observed discordances and found that approximately one half resulted from out-of-date classifications or submission errors, whereas the remainder were likely expert judgment differences regarding the strength or quality of particular scientific evidence. We were pleased that all but one laboratory responded collaboratively to requests for detailed information about their classifications—despite those requests coming directly from a commercial competitor (Invitae). This process of identifying and reconciling differences was made possible by shared data in a central and unrestricted public database (ClinVar).

Our findings might at first seem to be at odds with other studies that compared variant classifications. A study by Vail et al^42^ compared the interpretation of approximately 2,000 *BRCA1/2* variants among a number of public databases and found greater discordance than we report. The methodology of the study by Vail et al was significantly different from ours. Of importance, it incorporated data from research laboratories, older data, and data from curated literature databases that were not classified using modern clinical criteria.^8,43,44^ Furthermore, it counted differences that would not significantly change management, for example, VUS versus likely benign. Finally, it measured discordance only on a per-variant basis, not per-patient, which in our analysis was dramatically lower. These methodologic differences exaggerate the impact of discordance on clinical application.

Other studies have addressed variant classification concordance under different clinical circumstances. Maxwell et al^45^ studied their own application of ACMG guidelines to variants in a diverse set of hereditary cancer genes observed in patients and found an overall per-variant concordance with ClinVar that was high (95%) but lower than our corresponding result (98.5%). We examined all *BRCA1/2* variants in their study and found 100% concordance with our ClinVar data. Discordances that Maxwell et al found were in other cancer genes—only recently incorporated into tests—for which less information is generally available and thus discordance may indeed be higher than it is for *BRCA1/2*.

Separately, Balmaña et al^46,47^ examined variants in cancer genes other than *BRCA1/2* in the PROMPT registry.^48^ The authors found 19 unique variants, which represent 57 of 603 comparable test findings (9.5%), that had two or more significantly different interpretations in ClinVar. The authors concluded that “conflicting interpretation … is frequent and may have implications for medical management.”^48(p 46)^ We examined current (September 30, 2016) ClinVar entries for all 19 of these variants and found that six had discordant interpretations only from a nonclinical source (most commonly, OMIM^49^), whereas all clinical laboratories, in fact, agreed with each other.^47^ One variant was no longer discordant after a 1-year-old, but more recently submitted, reclassification. Two low-risk variants had discordance that was attributable to the fact that nomenclature and classification criteria for such variants are not standardized under current ACMG guidelines, yet most laboratories still agreed. We count 10 variants from Balmaña et al, representing 2.2% of findings (13 of 603), having a clinically substantial discordance between clinical testing laboratories, 4.3-fold fewer than the 57 of 603 they report. Some of this remaining discordance in non-*BRCA1/2* genes seems to be attributable to factors we describe above for *BRCA1/2*, for example, older data, although, unfortunately, Balmaña et al^46^ did not contact submitting laboratories to understand the basis of discordance as we did.

In another study, the National Institutes of Health–funded Clinical Sequencing Exploratory Research consortium performed an experiment in which 99 variants in various genes—biased toward relatively challenging cases—were classified by up to nine laboratories.^50^ Although many classification differences were observed, only a fraction would change management, and only five variants in *BRCA1*/*2* were included. Of importance, the authors found that sharing classifications among laboratories, thus identifying discordances, enabled discussions that resolved many of the differences and contributed to an overall higher quality than any one laboratory could achieve alone.

Our study and those mentioned above highlight important best practices in the use of public databases. Although variant classifications from all sources are valuable and important to centralize and share, database users must apply good judgment and quality control. They must pay attention to dates, as variant classifications can become outdated, for example, when new scientific evidence is published. Moreover, users must consider whether a classification originates from a clinical laboratory that rigorously follows guidelines-based classification procedures or from a submitter who may have applied a different standard. Finally, database users must evaluate the underlying scientific evidence for each classification, just as they do when considering variant classifications in any publication. At present, only two of the laboratories included in this study—GeneDx and Invitae— provide the evidence that supports the classification of specific variants in their ClinVar submissions, a situation that we hope will change. Other laboratories include evidence only in patient reports, but these are not broadly available for both logistical and patient privacy reasons.

Although our analysis shows that clinically significant disagreements in *BRCA1/2* variant classification are infrequent, they are, of course, important to patients and clinicians. We believe it is essential for the genetics community to resolve these differences collaboratively, as is standard practice in other areas of oncology, to deliver the best possible patient care.^21-23,38-41,50^ Our study supports others in demonstrating that collaborative interaction among laboratories improves the quality of clinical testing.^39-41,50^ Unlike proprietary databases, ClinVar is freely open to all and makes such collaboration possible on a global scale. Moreover, ClinVar enables independent assessment of variant classification accuracy and consistency, as exemplified by this study. Although laboratories with proprietary databases have made claims of superior accuracy, such claims are not subject to detailed and ongoing independent review.^14,17^ Indeed, our observation of high concordance across laboratories calls into question some of those claims. We note that semipublic databases with restrictive licensing terms, such as BRCA Share,^51-53^ can present many of the same challenges that are encountered with proprietary databases—for example, license restrictions prevented the consideration of such data in this study. We also note that patient registries, including PROMPT, although highly valuable for other reasons, do not address the needs that ClinVar does.^14,21^

For these reasons, the open sharing of deidentified variant classifications is recommended by the AMA, ACMG, National Society for Genetic Counselors, and other professional societies. In collaboration with international groups, the National Institutes of Health has recently funded initiatives, including ClinGen^21,22^ and the BRCA Exchange,^7^ that leverage ClinVar, the literature, and other resources, to share, compare, and reconcile variant classifications, thus continually improving this important aspect of precision oncology. However, at least one major laboratory, Myriad Genetics, has revised its terms of service to prohibit ordering clinicians from sharing deidentified variant classifications,^54^ which is how SCRP data used in this study were obtained. Myriad Genetics has also historically resisted requests from patients for their unreported benign variants, prompting legal action by the American Civil Liberties Union.^55^ Such restrictions will make ongoing comparative analyses impossible. We hope that our study illustrates the importance of open and unrestricted genetic data sharing via ClinVar and the value of supporting this critical initiative.
